# A comparative analysis of insertional effects in genetically engineered plants: considerations for pre-market assessments

**DOI:** 10.1007/s11248-014-9843-7

**Published:** 2014-10-26

**Authors:** Jaimie Schnell, Marina Steele, Jordan Bean, Margaret Neuspiel, Cécile Girard, Nataliya Dormann, Cindy Pearson, Annie Savoie, Luc Bourbonnière, Philip Macdonald

**Affiliations:** 1 Plant and Biotechnology Risk Assessment Unit, Canadian Food Inspection Agency, 1400 Merivale Road, Ottawa, ON K1A 0Y9 Canada; 2 Animal Feed Division, Canadian Food Inspection Agency, 59 Camelot Drive, Ottawa, ON K1A 0Y9 Canada; 3 Food Directorate, Health Canada, 251 Sir Frederick Banting Driveway, Ottawa, ON K1A 0K9 Canada; 4 Plant Biosafety Office, Canadian Food Inspection Agency, 59 Camelot Drive, Ottawa, ON K1A 0Y9 Canada; 5 Office of the Chief Science Operating Officer, Canadian Food Inspection Agency, 1400 Merivale Road, Ottawa, ON K1A 0Y9 Canada

**Keywords:** Insertional effects, Novel trait, Unintended trait, Genetic engineering, Conventional breeding, Pre-market assessment

## Abstract

During genetic engineering, DNA is inserted into a plant’s genome, and such insertions are often accompanied by the insertion of additional DNA, deletions and/or rearrangements. These genetic changes are collectively known as insertional effects, and they have the potential to give rise to unintended traits in plants. In addition, there are many other genetic changes that occur in plants both spontaneously and as a result of conventional breeding practices. Genetic changes similar to insertional effects occur in plants, namely as a result of the movement of transposable elements, the repair of double-strand breaks by non-homologous end-joining, and the intracellular transfer of organelle DNA. Based on this similarity, insertional effects should present a similar level of risk as these other genetic changes in plants, and it is within the context of these genetic changes that insertional effects must be considered. Increased familiarity with genetic engineering techniques and advances in molecular analysis techniques have provided us with a greater understanding of the nature and impact of genetic changes in plants, and this can be used to refine pre-market assessments of genetically engineered plants and food and feeds derived from genetically engineered plants.

## Introduction

Genetic engineering is frequently used to introduce new DNA sequences that contain one or more genes into plants, leading to the development of plants that exhibit novel traits. Given the complexity of plant cells and the current limitations of genetic engineering, unintended traits may result from genetic engineering in addition to the novel trait. Unintended traits are phenotypic changes in the plant that can materialize as a consequence of genetic changes such as DNA insertions, deletions and rearrangements, all of which can take place during genetic engineering, and the associated tissue culture process. These changes may include the introduction of a new plant characteristic, loss of a previously expressed plant characteristic or expression of a characteristic that is outside the range of what is typically observed in the plant species. The safety of novel traits intentionally introduced into plants during development is assessed during the pre-market assessment of genetically engineered plants, however, unintended traits are by their nature unexpected, making it difficult to test for them directly.

In most countries, a comparative approach used in the pre-market assessment of genetically engineered (GE) plants and foods and feeds derived from GE plants (hereafter, GE plants, foods and feeds) provides a structure that enables the identification and evaluation of unintended traits. The comparative approach was initially developed for the pre-market assessment of genetically engineered organisms in 1986, and has been adopted and used by national regulatory bodies for over 15 years to conduct pre-market assessments on more than a hundred GE plants, foods and feeds, many of which have become integral parts of the agricultural production systems and food and feed chains of more than 20 countries (Codex Alimentarius Commission 2003; OECD 1986, 1992, 2003). The comparative approach is important because it recognizes that conventional plant varieties, foods and feeds have a history of safe use, and allows the pre-market assessment of the GE plant, food or feed to focus on differences between the GE plant, food or feed and a counterpart in order to determine potential safety concerns. The counterpart is typically the isogenic line closest to the GE plant with an established history of safe use, which is usually a conventional plant (i.e., not genetically engineered). This approach acknowledges the pre-existing nutritional profile, toxins, allergens and anti-nutrients of conventional plants, foods and feeds as well as the impact of the conventional plant and its associated agricultural practices on the environment.

Pre-market assessments, based on the comparative approach, function by defining the novel trait expressed in the GE product, identifying any unintended traits, and assessing the potential of these traits to adversely affect the environment, or impact the safety and/or nutritional quality of the food or the safety and/or efficacy of the feed derived from the GE plant. To accomplish this, pre-market assessments include a molecular characterization of the GE plant that considers the inserted DNA, expressed gene products and their impact on the GE plant, and a comparison of the composition and phenotype of the GE plant to that of a counterpart. The choice of which components and phenotypic characteristics to examine is, in part, determined by the nature of the novel trait, but it is generally broader to allow for the identification of unintended traits as well.

One source of unintended traits in GE plants is insertional effects, which are the changes to the transformed plant’s genome that result from the process of inserting DNA by genetic engineering (Kessler et al. 1992; König et al. 2004). In addition to the intended DNA, in many cases additional DNA is also inserted. The additional DNA can originate from either the plant genome, the DNA being inserted or it can simply be filler DNA of unknown origin (Forsbach et al. 2003; Kim et al. 2003; Kohli et al. 1999; Krysan et al. 2002; Makarevitch et al. 2003; Sallaud et al. 2003; Windels et al. 2003). Also common are small deletions in the plant genomic DNA flanking the site of insertion. These deletions are typically less than 100 bp, although larger deletions are also occasionally observed (Forsbach et al. 2003; Kim et al. 2003; Krysan et al. 2002; Makarevitch et al. 2003). Large scale rearrangements may also occur during genetic engineering. *Agrobacterium tumefaciens*-mediated transformation and biolistics-mediated transformation are two commonly used approaches for creating genetically engineered plants. Both of these approaches can result in complex genetic inserts containing multiple copies and/or re-arrangements of both the DNA intended to be inserted and the host plant DNA (Dan and Ow 2011; Stewart et al. 2011). For instance, in arabidopsis (*Arabidopsis thaliana*) transformed using *A. tumefaciens*, chromosomal translocations have been documented, where the flanking genomic DNA on either side of the inserted DNA mapped to two different chromosomes (Forsbach et al. 2003; Nacry et al. 1998). These insertional effects can alter patterns of gene expression in the plant or change the nature of RNA and/or proteins expressed by endogenous genes, either of which could result in an unintended trait in the plant.

The comparative approach to the pre-market assessment of GE plants, foods and feeds continues to be valid. However, there is now increased familiarity with genetic engineering techniques, as well as a growing body of scientific research both on the process of genetic engineering and on GE plants, foods and feeds themselves. In addition, advancements in molecular analysis techniques, particularly high-throughput sequencing and global profiling technologies, have given us an unprecedented understanding of the nature and the impact of genetic changes in plants. This raises the question of whether the comparative approach may be further refined.

It is, therefore, an opportune time to review and contextualize our understanding of insertional effects and their potential to result in unintended traits and to investigate how these insertional effects may be best evaluated within the current comparative approach to pre-market assessments. A simultaneous review of genetic changes that occur spontaneously as well as those that occur during conventional breeding will provide a useful comparison for insertional effects in keeping with the comparative approach to pre-market assessments. If insertional effects are found to be similar to other genetic changes that occur in plants, they should present a similar level of risk. It would then be reasonable to re-evaluate the information required for the comparative pre-market assessment of GE plants, foods and feeds.

## Scope

This review will be limited to a discussion of potential unintended traits resulting from insertional effects. Other potential sources of unintended traits exist, including pleiotropic effects, which occur when a gene within the inserted DNA sequence confers multiple traits, and position effects, which are variations in the expression of genes within the inserted DNA that are dependent on the site of insertion.

## Insertional effects and unintended traits

Although it is sometimes assumed that insertional effects are in and of themselves an indication that a GE plant will display an unintended trait, this is not an accurate conclusion. In a T-DNA insertional mutagenesis study in rice (*Oryza sativa*), examination of 22,665 field-grown T1 lines identified only 4,065 lines with a visible mutant trait (Chern et al. 2007). A study by El Ouakfaoui and Miki (2005) investigated the impact of the insertion of two marker genes on global gene expression profiles in four arabidopsis lines. In three independent experiments, reproducible changes in gene expression were not observed for any genes (with the exception of the introduced genes themselves) demonstrating that genes can be inserted without altering the global gene expression profile (El Ouakfaoui and Miki 2005).

Since not all insertional effects will result in an unintended trait, the question then becomes: what factors will determine whether an insertional effect will cause an unintended trait in a GE plant? In most cases, the DNA will need to be inserted within or near to an endogenous gene. The likelihood of this occurring will vary from species to species based on genome composition. For instance, gene rich regions are expected to account for 17 % of the maize (*Zea mays*) genome with an average gene density of 1 gene per 40 kb (Barakat et al. 1997; Messing et al. 2004). In contrast, in rice, gene rich regions account for 24 % of the genome and the average gene density is one gene every 4 to 5 kb (Barakat et al. 1997; Yu et al. 2002). Although some studies have suggested that transgene integration in *A. tumefaciens*-mediated transformation is biased towards actively transcribed genes, results of other studies indicate that at least some of this perceived insertion site bias is due to selection in insert identification (Francis and Spiker 2005; Kim and Gelvin 2007).

If the insertion occurs within or near to an endogenous gene or regulatory element, either the expression of associated endogenous gene(s) or the nature of the RNA(s) and/or protein(s) produced must then be affected in order for an insertional effect to result in an unintended trait. The expression of an endogenous gene can change if insertions, deletions and rearrangements within the gene render it non-functional, completely abolishing expression, or alter its pattern or level of expression. Regulatory elements within the inserted DNA can also influence the expression of nearby endogenous genes. This has been exploited in activation tagging, where DNA is inserted that contains a moderate to strong enhancer or promoter, such as the CaMV 35S promoter, with the goal of transcriptionally activating nearby genes (Chern et al. 2007; Jeong et al. 2002). In one such study, it was found that the average distance between the inserted enhancer and the activated gene was 4.4 kb, although this distance could be as great as 8 kb (Ichikawa et al. 2003). The second case, wherein the expressed sequence of the RNA(s) and/or protein(s) of an endogenous gene is changed, could occur by a number of mechanisms, for example due to insertions, deletions and/or rearrangements within the gene resulting in it being truncated, alternatively spliced, or if a novel chimeric gene is created.

Even if there are changes in the expression of an endogenous gene or the nature of the gene product that is expressed, these will not necessarily result in an unintended trait. The levels of expression of a given gene will vary based on genetic background and environment, so any changes within this range of expression may fall within normal variation and therefore not be biologically significant. For example, the expression of thousands of plant genes are frequently altered in response to stress (El Ouakfaoui and Miki 2005; Kawaura et al. 2006; Zheng et al. 2010). Even in the absence of stress, many genes are differentially expressed in plants grown at different locations and under different environmental conditions (Barros et al. 2010).

Furthermore, in cases where the function of the endogenous gene is compromised by insertional effects, it may still not result in an unintended trait because of the high level of gene redundancy in plant genomes. Genomic analysis of arabidopsis and rice has revealed gene redundancy throughout approximately one-third to one-half of the genome (Goff et al. 2002; The Arabidopsis Genome Initiative 2000; Yu et al. 2002). The buffering effect of duplicate genes is a legacy of polyploidization, which can be found in the ancestry of most plant species (Adams and Wendel 2005; Comai 2005). Duplicate copies of a damaged or aberrantly expressed gene can mask potentially detrimental phenotypes. For example, in 4,000 greenhouse-grown arabidopsis lines with insertions of a transposon in gene-coding regions, only 139 showed a visible mutant phenotype (Kuromori et al. 2006). In another example, only about 2 % of a mutant soybean (*Glycine max*) population generated by fast neutron (FN) radiation showed an abnormal visual phenotype (Bolon et al. 2011).

Finally, changes to the nature of the RNA and/or protein that is expressed from an endogenous gene may also fail to result in an unintended trait due to the RNA and protein quality control systems active in plant cells. These quality control systems actively target aberrant RNA and protein for degradation (Buchberger et al. 2010; Doma and Parker 2007), minimizing their impact on the plant phenotype.

In conclusion, the relationship between genotype and phenotype in plants is complex, and the role of the environment cannot be ignored. In many ways, plants are buffered against the consequences of genomic changes by the high level of gene redundancy in plant genomes and by quality control systems active in plants. All of these factors influence whether or not an insertional effect will produce an unintended trait. Therefore, while insertional effects, i.e. the changes to the transformed plant’s genome that result from the process of inserting DNA by genetic engineering, are unavoidable during genetic engineering, unintended traits are not always a consequence of these genetic changes. This conclusion is supported by the fact that detailed review of the nutritional composition of over 100 GE plants in the United States and Canada failed to identify adverse effects of genetic engineering on the composition of these plants (Price and Underhill 2013).

## Genetic changes similar to insertional effects occur in plants

Plant genomes change over time in response to natural selection and genetic drift. Those spontaneous genetic changes that confer traits that better allow a plant to survive and adapt are maintained by the species. The genomes of crops have additionally been shaped by humans through conventional breeding, through the selection of desired traits that have arisen spontaneously or been created through mutagenesis. Despite the genetic changes associated with conventional breeding, there is a history of safe use of conventional crops. It is therefore useful to determine whether the genetic changes that occur in plants, either spontaneously or through conventional breeding, are similar to insertional effects as a consequence of genetic engineering.

### The movement of transposable elements

The impact of the insertion of DNA through genetic engineering is probably most closely paralleled by the movement of transposable elements. Transposable elements can be found in the genomes of all plants. In arabidopsis, they account for at least 10 % of the genome, in rice at least 24.9 %, and in maize at least 57 % (Messing et al. 2004; The Arabidopsis Genome Initiative 2000; Yu et al. 2002). Typically ranging in size from hundreds to thousands of base pairs and, for autonomous elements, containing one or more open reading frames, the impact of their insertion into novel genomic loci is similar to that of the insertion of DNA via genetic engineering (Bennetzen 2000). In fact, many transposable elements were first identified by the mutant traits they generated and this phenomenon has been exploited in large scale insertional mutagenesis studies (Greco et al. 2001; Kuromori et al. 2006; Walbot 2000). Like DNA insertions via genetic engineering, if a transposable element inserts within or near a plant gene, it can inactivate the gene, alter its level or pattern of expression, or alter the nature of the RNA and/or protein that is expressed by that gene (Casacuberta and Santiago 2003). This can lead to the expression of novel traits. For instance, transposition of a *doppia* transposable element resulted in the formation of the *R*-*r:std* allele in maize, within which the *R*-*s* subcomplex is expressed in the aleurone layer of seeds, leading to its pigmentation (May and Dellaporta 1998; Walker et al. 1995). New cultivars of fruit have arisen from sports where the underlying genetic change is the insertion of a transposable element in close proximity to a plant gene. Research on grape (*Vitis vinifera*) berry colour has identified a connection between a lack of anthocyanin pigment (i.e. white berry) phenotype and the insertion of *Gret1*, a 10 422 bp long retrotransposon, in the promoter region of *VvmybA1*, a gene located in the berry colour locus that encodes a transcription factor that induces the anthocyanin pathway (Kobayashi et al. 2004). Insertion of this retrotransposon separates the *VvmybA1* coding sequence from its promoter and impairs its transcription, thus preventing anthocyanin production. The opposite effect has been observed in blood oranges (*Citrus sinensis*), where the insertion of a Copia-like retrotransposon adjacent to the *Ruby Myb* gene gave rise to the induction of the anthocyanin biosynthesis pathway (Butelli et al. 2012).

As is the case with the insertion of DNA through genetic engineering, the movement of transposable elements is also associated with insertions, deletions and rearrangements. Although movement of transposable elements between plant species has been reported (El Baidouri et al. 2014), the movement of transposable elements within a genome is much more common and would make a greater contribution to genetic change. DNA transposons often leave behind a footprint at the site of their excision, which may include small deletions, inversions, and insertions, including sequence from the transposable element itself (Bennetzen 2000; Rinehart et al. 1997). Rearrangements can result from aberrant transposition, which is known to occur with DNA transposons such as the *Ac*/*Ds* family (Gray 2000). As with the insertion of DNA through genetic engineering, these insertions, deletions and rearrangements that are associated with the movement of transposable elements can alter the nature and/or regulation of plant genes. In many cases, transposable element activities have played a significant part in plant evolution (Lisch 2013). For example, an in-depth analysis of transposable elements in sequenced crop genomes identified 51 transposable element-induced phenotypic changes associated with domestication or diversification of cultivated plants (Vitte et al. 2014).

The extent of movement of transposable elements in cultivated plants is not yet fully known. Most transposable elements are likely inactive, but some are known to be active in certain tissues or during certain developmental stages (Bennetzen 2000; Grandbastien 1998). Transposable elements may also become activated in response to stress, major genomic rearrangements, such as chromosome breaks, polyploidization, and hybridization, as well as during protoplast isolation, and cell and tissue culture (Bennetzen 2000; Grandbastien 1998; Parisod et al. 2010; Shan et al. 2005; Wang et al. 2010; Zou et al. 2011). In contrast to sporadic activation, some transposable elements, such as those identified in rice, appear to be continuously active under normal conditions of growth (Moon et al. 2006; Naito et al. 2006). Estimates of the frequency of movement of transposable elements vary. For example, approximately one new insertion of the *nDart* transposable element was found in every four plants in a study of backcrosses with the rice cultivar Hoshinoyume (Fujino et al. 2005). In contrast, a single rice line was reported to accumulate dozens of de novo *mPing* transposable elements per generation (Naito et al. 2006).

### Repair of double-strand breaks by non-homologous end-joining

Double-strand breaks may be caused by physical stress on chromosomes, DNA replication across a nick, DNA crossing over during homologous recombination, or by reactive oxygen species, which are produced by the plant cell as a by-product of metabolism, in response to pathogen attack, or by exposure to environmental pollutants, ionizing radiation and ultraviolet light.

The main pathway for repair of double-strand breaks in plants is non-homologous end-joining (NHEJ). NHEJ is an error-prone recombination pathway, and deletions, insertions and rearrangements are often observed at the repair site where the two DNA ends have been rejoined (Gorbunova and Levy 1999). These are similar to the deletions, insertions and rearrangements that are often observed at the site of DNA insertion in plants having undergone genetic engineering. As with genetic engineering, if these deletions, insertions and rearrangements involve endogenous genes, the nature and/or regulation of these genes can be affected.

A hallmark of NHEJ repair sites is the presence of short repeats, sometimes referred to as microhomologies, that are typically 1–8 bp in length (Gorbunova and Levy 1999; Morita et al. 2009). These will be present when the repair is mediated by annealing of the exposed ends at these repeats. Interestingly, microhomologies are also often observed at sites where DNA has been inserted by genetic engineering, between the inserted DNA and the flanking plant genome sequences (Gorbunova and Levy 1999; Somers and Makarevitch 2004). This is because both *Agrobacterium*-mediated and particle bombardment transformation methods rely on the plant’s DNA repair pathways, in particular NHEJ, to introduce the DNA, although the exact mechanisms have not yet been fully elucidated (Makarevitch et al. 2003; Mayerhofer et al. 1991; Somers and Makarevitch 2004; Takano et al. 1997). Furthermore, the footprints created by excision of DNA transposons also contain these microhomologies because, once again, NHEJ is involved in repairing the double-strand break caused by excision (Huang and Dooner 2012; Rubin and Levy 1997). The common mechanism that underlies the generation of the insertions, deletions and rearrangements found at sites of DNA insertion via genetic engineering, double-strand break repair and DNA transposon excision is evidence that these insertional effects are not unique to genetic engineering.

The generation of double-strand breaks and their repair is also a key feature of conventional breeding. As the phylogenetic distance between a cultivated crop and its wild relative becomes wider, it becomes more difficult to create hybrids and introgress desired traits. In such cases, traits can be introgressed through chromosomal translocations. Infrequently, such translocations may occur spontaneously, but in many cases they must be induced, for instance by using ionizing radiation to cause double-strand breaks (Fedak 1999; Friebe et al. 1996). Ionizing radiation is also used to induce double-strand breaks in order to create mutations. The resulting mutants typically have deletions ranging in size from tens to millions of base pairs, as well as rearrangements, including inversions and chromosomal translocations (Cecchini et al. 1998; Li et al. 2001; Morita et al. 2009; Nambara et al. 1994; Sato et al. 2009; Schuermann et al. 2005; Shirley et al. 1992; Sun et al. 1992; Yan et al. 2007). Again, microhomologies are often observed at repair sites. A comparison of genetic changes found at sites of double-strand break repair resulting from genetic engineering, cleavage with a restriction endonuclease and mutagenesis with gamma irradiation can be found in Table 1, further highlighting their similarity.

**Table 1:** A comparison of genetic changes found at sites of double-strand break repair resulting from genetic engineering (Forsbach et al. 2003), cleavage with a restriction endonuclease (Salomon and Puchta 1998) and mutagenesis with gamma irradiation (Morita et al. 2009)

	Microhomology		Insertions		Deletions		Rearrangements	
	Number of sites/total sites analyzed	Size range (bp)	Number of sites/total sites analyzed	Size range (bp)	Number of sites/total sites analyzed	Size range (bp)	Number of sites/total sites analyzed	Size range (bp)
Forsbach et al. (2003)	66/186^a^	1–8	109/186^a^	1–1,700	64/73	1–300	3/73	20, translocations
Salomon and Puchta (1998)	13/17	1–15	18/28	4–2,355	27/28	1–1,322	0/28	N/A
Morita et al. (2009)	16/21	1–8	1/21	1	21/21	1–129,700	2/21	1,284,800; 3,208,500

The number of sites at which microhomologies, insertions, deletions and rearrangements were identified is shown versus the total number of sites analyzed, as well as the size range of each

^a^Sequences at the left and right borders of the T-DNA insertions were analyzed separately

### Intracellular transfer of organelle DNA

The presence of non-coding sequences in plant nuclear genomes that are homologous to plastid and mitochondrial sequences (nuclear organelle DNAs) suggests that organelle DNAs have repeatedly been inserted into the nuclear genome. In some cases it is clear that organelle DNA was inserted intact in the nuclear genome while in other cases rearrangements are apparent. In rice and arabidopsis, approximately 25 % of nuclear organelle DNAs occur within genes (Richly and Leister 2004), and in some cases sequences have become functional parts of coding regions (Noutsos et al. 2007). Once again, microhomologies found at the sites of insertion of nuclear organelle DNAs indicate that these sequences are inserted by NHEJ, and consistent with this mechanism, deletions and insertions were observed at sites of insertion (Huang et al. 2004). The insertion of organelle DNA into the nuclear genome is therefore similar to the insertion of DNA during genetic engineering. Like the insertion of DNA through genetic engineering, the nuclear organelle DNAs have the potential to alter the level or pattern of expression of endogenous genes or alter the nature of the RNA and/or protein expressed by endogenous genes.

The frequency with which organelle DNA is transferred to the nucleus is not well characterized, but there is evidence in tobacco (*Nicotiana tabacum*) that novel nuclear organelle DNAs are formed under typical conditions of growth. Huang et al. (2003) transformed the tobacco chloroplast genome with a nucleus-specific marker gene and found that the marker transferred to the nucleus at a rate of 1 in every 16,000 progeny. This number is presumed to be an underestimation of the true transfer rate, since it required transfer of the marker gene in a functional state. Wang et al. (2012) further demonstrated that the rate of transfer of DNA from the chloroplast to the nucleus increased up to tenfold in response to mild temperature stresses.

### Other sources of genetic change in plants due to inserted DNA

There are additional possible sources of genetic change in plants that are similar to insertional effects. These changes have been previously reviewed (Bock 2010; Liu et al. 2012) and while it is not the intent of this publication to review all sources of genetic change in plants in detail, a few examples will be provided. Pararetroviruses are double stranded DNA viruses that infect plants. Although these viruses lack integrase enzymes, endogenous pararetrovirus DNA had been found in various plant genomes such as rice, tobacco, tomato (*Solanum lycopersicum*), potato (*Solanum tuberosum*), banana and plantain (*Musa* spp.), and petunia (*Petunia* spp.) (Liu et al. 2012; Staginnus et al. 2007; Staginnus and Richert-Pöggeler 2006). The mechanism by which these sequences are inserted into plant genomes has not been elucidated but is considered to likely involve NHEJ (Liu et al. 2012). Another example is processed pseudogenes. Pseudogenes are nonfunctional genomic sequences with significant sequence similarity to functional RNA or protein-coding genes (Vanin 1985). Processed pseudogenes, which are derived from retrotransposition events where double-stranded cDNAs derived from reverse transcription events are integrated into the genome (Zou et al. 2009), represent a source of inserted DNA in plants. Finally, *A. tumefaciens* and closely related bacteria are capable of transferring bacterial genes into plant genes, and this ability long predates genetic engineering (Gelvin 2000).

As we have illustrated here, the insertion of DNA via genetic engineering is similar to the movement of transposable elements and the transfer of organelle DNA and other DNA sequences to the nucleus; and the insertions, deletions and rearrangements that are often observed at the site of DNA insertion by genetic engineering are no different than those observed at sites where double-strand breaks have been repaired, which is reflective of their common underlying mechanism. This information demonstrates, therefore, that insertional effects are similar to other genetic changes that occur spontaneously in plants and during conventional breeding. By virtue of their association with the novel trait, insertional effects will be present throughout a variety as long as the novel trait is maintained. In contrast, spontaneous genetic changes will arise in a single plant and are less likely to become established in a variety. Similar to insertional effects, with conventional breeding many of the genetic changes will be closely linked to the desired trait that is being selected, and as a result these will be present throughout a variety as long as that desired trait is maintained. Therefore the strongest parallel can be drawn between the insertional effects of genetic engineering and the genetic changes associated with conventional breeding.

## Other genetic changes also occur in plants

In the previous section, we considered those genetic changes that are most similar to insertional effects. However, it is also important to consider other types of genetic changes that occur both spontaneously and as a result of conventional breeding in plants, because it is within this background of genetic change that insertional effects occur. Although these other genetic changes may not be similar in nature to insertional effects, they have the potential to be similar in impact.

### Spontaneous genetic change

Mutations occur spontaneously in all living organisms. For instance, in arabidopsis, spontaneous mutations occur at a rate of 7 × 10^−9^, which translates into 1.75 mutations per generation per diploid plant (Ossowski et al. 2010; Weber et al. 2012). If this rate of genetic change were consistent in plants with larger genomes, the number of mutations per generation would be correspondingly larger. For example, based on the genome size of soybean (Schmutz et al. 2010), more than 10 mutations per generation would be expected to spontaneously occur. This level of mutation is consistent with reports that single-nucleotide-polymorphisms (SNPs) were detected every 48–2,000 base pairs in wheat (*Triticum aestivum*), soybean, and maize, with coding regions containing fewer SNPs than noncoding regions (Weber et al. 2012). These mutations may occur during DNA replication, either due to mispairing, which results in single base substitutions, or strand slippage, which results in small insertions and deletions. Spontaneous lesions can also form, where bases are either lost or modified, causing them to mispair and again leading to single base substitutions. We discussed already the ability of environmental pollutants, ionizing radiation and ultraviolet light to cause double-strand breaks. These can also alter the structure of DNA directly, leading to single base substitutions.

Spontaneous genetic changes can also occur during homologous recombination, which, like NHEJ, can repair double-strand breaks, but also introduces genetic variation during meiosis. During homologous recombination, copy number variants can result from misalignment between repeated sequences on homologous chromosomes, leading to unequal crossing over. In arabidopsis, copy number variants were found to occur at a rate of 1–3 × 10^−6^ for a given gene cluster, suggesting that a copy number variant could be formed as often as 1 in every 700 seeds (Gaut et al. 2007; Jelesko et al. 2004). Intrachromosomal recombination can also occur, and the outcome can include sequence deletions, inversions, and gene conversion. Recombination between homologous sequences located at different genomic locations can lead to large chromosomal rearrangements, including translocations (Gaut et al. 2007).

In addition to the impacts of transposable elements on plant genomes already discussed, transposable elements can further alter plant genomes in ways for which there is no parallel observed during genetic engineering. For example, transposable elements will occasionally amplify genomic DNA sequences (Bennetzen 2000). This can lead to increases in gene copy number if the genomic DNA sequence contains a gene. Such amplifications can give rise to novel traits, as was observed for the tomato variety Sun1642, for which an elongated fruit trait has been attributed to the duplication of the gene *SUN* during the movement of a retrotransposon (Xiao et al. 2008). In addition, the capture of genes and gene fragments by transposable elements can lead to the creation of new genes through exon shuffling (Bennetzen 2005). In rice, many of the *Mutator*-like DNA elements (MULEs) contain gene fragments, often from different chromosomal loci, and there is evidence that at least some of these gene fragments are being expressed (Jiang et al. 2004). Likewise in soybean, the *Tgm*-*Express1* transposon (of the CACTA super family) carries multiple captured gene fragments. The presence of this transposon in Intron 2 of the flavanone 3-hydroxylase gene (*F3H*) (i.e. the *wp* mutant flower colour gene) results in the generation of several chimeric transcripts from alternative splicing of the pre-mRNA (Zabala and Vodkin 2007). The expression of these transcripts (including a transcript identical to that of the wild type gene) most likely contributes to the resultant mutant (i.e. pink) flower phenotype.

Finally, on a larger scale, plants may sometimes loose or gain whole chromosomes. This occurs during cell division, when chromosomes are not distributed properly between cells. Similarly, mitotic or meiotic failures or the fusion of unreduced gametes can lead to whole genome duplications. There is evidence that most plants have undergone at least two, and as many as three to four, whole genome duplications at some point in their evolution, suggesting that this process has had a major impact on plant evolution (Adams and Wendel 2005; Jiao et al. 2011; Leitch and Leitch 2008). Whole genome duplications are typically followed by a series of rearrangements, including extensive sequence loss, homologous recombination, translocations, as well as adjustments to gene expression (Adams and Wendel 2005). Sorghum (*Sorghum bicolour*) and rice have largely collinear genomic maps with maize, permitting comparison of their chromosomal arrangements. A comparison of select genomic regions from these three species suggests that at least 50 % of duplicate genes in maize were lost following polyploidization (Lai et al. 2004).

Clearly there are a number of mechanisms at work in plants that can bring about genetic changes. The cumulative impact of these genetic changes can be illustrated by comparative genomic analyses, which have demonstrated that plant genomes are remarkably diverse even within a given species. The maize genome, in particular, is known to be highly polymorphic. In a survey of the 3′ ends of 18 genes within 36 elite maize inbred lines, SNPs were found to occur on average every 60.8 bp and small insertions and deletions every 126 bp, ranging in size from 1 to 400 bp (Ching et al. 2002). Larger scale rearrangements are also common between individuals within a species, with genes found in different locations, different copy numbers or even absent in some individuals and present in others (i.e. presence/absence variation). A comparative genomic hybridization analysis of 19 maize genotypes and 14 teosinte genotypes using a custom microarray with 32,487 maize genes identified 1,065 genes with copy number variation and 2,824 genes that were present in some genotypes and absent in others (Swanson-Wagner et al. 2010). Similarly, a comparison of 2,094 contigs from the rice varieties Nipponbare and 93-11, which accounted for approximately 10 % of the whole genome, revealed 156 genes that were asymmetrically located, 91 with copy number variation, and 82 genes that were present in one variety and absent in the other (Ding et al. 2007). Recently, the use of whole genome sequencing has revealed that the potato, maize, and soybean genomes possess dozens to thousands of presence/absence variations between individual varieties of their respective species (Lai et al. 2010; Lam et al. 2010; McHale et al. 2012; Potato Genome Sequencing Consortium 2011).

Furthermore, gene movement is extensive in large plant genomes such as wheat, barley (*Hordeum vulgare*), and their relatives. Comparisons between syntenic (i.e. collinear) regions of the wheat, *Brachypodium*, rice, and sorghum genomes show that the movement (or duplication) of syntenic genes to non-syntenic positions are most likely resultant of transposable element activity and double-strand break repair (Wicker et al. 2010). These mechanisms frequently result in the creation of a pseudogene that may or may not be functional in its new position in the genome. However, if the non-syntenic gene maintains the functional activity of its homolog (i.e. the gene from which it was duplicated), and the homolog is somehow lost, the non-syntenic gene may be selected for and maintained at its new position, further eroding collinearity between related plant species (Wicker et al. 2010, 2011).

Some types of genetic change, such as DNA replication errors, spontaneous lesions, and the formation of copy number variants during recombination, constantly occur in plants. In contrast, the amount of DNA damage from exposure to external mutagens is highly dependent on environmental conditions, and the activity of transposable elements will similarly be dependent on a number of internal and external factors, as already discussed. While large changes such as polyploidization and other ploidy changes occur infrequently, they do illustrate the remarkable ability of plants to undergo large-scale genomic changes. The frequency of polyploidization events in the evolutionary history of flowering plants suggests that large-scale genomic changes in some cases offer a selective advantage.

### Conventional breeding

The most commonly applied conventional breeding technique is the introgression of a desired trait either from another individual within the same species, via intraspecific crossing, or from another species, typically a wild relative, via interspecific crossing. Genes for pest and disease resistance are more commonly targeted from these wild relatives. A study of 19 important commercial crops revealed evidence of introgression from 60 wild relatives into 13 of these crops (Hajjar and Hodgkin 2007). Another study on the use of introgression of disease resistance genes into wheat reported that six different genera have been used successfully as sources of these genes in wheat (Jones et al. 1995). Using these approaches, it is unlikely that only the gene responsible for the desired trait will be introduced into the final plant. Often, large amounts of DNA sequence surrounding the gene of interest will be transferred simultaneously, resulting in undesired traits being introduced along with the desired trait; this is known as linkage drag. Since some genes may be present in some individuals but not in others, intraspecific crosses have the potential to not only introduce novel alleles, but also novel genes. Linkage drag can be minimized by repeated backcrossing to eliminate more and more of the introgressed DNA sequence, but this process can be time-consuming, and it may not be possible to completely eliminate all of the undesired traits. For instance, the *Mi* locus in tomato introgressed from *S. peruvianum* can only be reduced to 650 kb and contains a number of genes, including a transposase, several with similarity to the arabidopsis transport inhibitor response-1 (*TIR*-*1*), and one with similarity to an arabidopsis transcription factor (jumonji) family protein (Seah et al. 2007). In another study, a wide variation was observed in the sizes of introgressed regions surrounding the *Tm*-*2* gene of *S*. *peruvianum*, which was introgressed independently intro various tomato cultivars, with one cultivar containing an entire chromosome arm (Young and Tanksley 1989).

As the phylogenetic distance between a cultivated crop and its wild relative becomes wider, it becomes more difficult to create hybrids and introgress the desired traits. Techniques such as embryo rescue, somatic hybridization and chromosome doubling may be employed in order to first establish viable hybrids. As already discussed, ionizing radiation may be used to create double-strand breaks in order to induce chromosomal translocations. Hybridization between distantly related species and chromosome doubling can have genome-wide consequences, including complete or partial elimination of the chromosomes of one of the two parents, and genome rearrangements, including translocations and recombination (Du et al. 2008; Liu and Li 2007). The resulting hybrids are often asymmetric, with the majority of the genome originating from one parent with single chromosomes or chromosome fragments belonging to the other parent (Du et al. 2008; Faure et al. 2002; Liu and Li 2007; Tu et al. 2009; Xu et al. 2007). Three recombinant inbred lines (RILs) derived from the asymmetric hybridization between rice and its wild relative *Zizania latifolia* contained less than 0.1 % of *Z. latifolia* DNA, and yet genomic variation (in the form of SNPs and small insertions and deletions) was observed at approximately 30 % of the loci examined (Wang et al. 2005). In addition, hybridization can sometimes result in the activation of transposable elements, the activity of which can further alter the genome, as already discussed (Shan et al. 2005; Wang et al. 2010; Zou et al. 2011).

Mutagenesis is another conventional breeding technique that creates novel genetic variation from which desired traits can be selected. The FAO/IAEA Mutant Variety Database (mvgs.iaea.org/AboutMutantVarities.aspx) currently lists 3,218 mutant plant varieties that are released worldwide. In these varieties, mutations were most frequently induced by ionizing radiation (predominantly using gamma rays and X-rays), although chemical mutagens were occasionally used (Ahloowalia et al. 2004). As already discussed, ionizing radiation can induce double-strand breaks. These breaks are repaired by NHEJ and often lead to deletions and rearrangements, but it can also alter bases leading to single base substitutions. For instance, gamma ray-induced single base substitutions in the rice *GA20ox*-*2* gene are responsible for the *sd1* mutation found in Japanese and American high-yielding semi-dwarf rice varieties that are widely commercialized (Ashikari et al. 2002). The popularity of chemical mutagens, particularly ethyl methanesulfonate (EMS), has increased in recent years. Chemical mutagens predominantly induce single base substitutions (Cooper et al. 2008; Greene et al. 2003; Olsen et al. 1993; Talamè et al. 2008; Uauy et al. 2009; Xin et al. 2008). EMS alters guanine residues such that they pair with thymine as opposed to cytosine. Following DNA repair, the G/C pair may be converted to an A/T (Greene et al. 2003). Other alkylating agents such as dimethyl sulphate and diethyl sulphate are expected to have similar effects (Hoffmann 1980).

Finally, in both conventional breeding and genetic engineering, plants can be regenerated from cell or tissue culture, and this laboratory technique can give rise to genomic level changes known as somaclonal variation. Somaclonal variation is both an unintended effect of cell and tissue culture and a potential source of novel traits for selection. Somaclonal variation can take the form of single base substitutions, insertions, deletions, rearrangements or changes in chromosome number (Jiang et al. 2011; Karp and Maddock 1984; Lee and Phillips 1987; van den Bulk et al. 1990). In addition, transposable elements may be activated by cell and tissue culture. For instance, in regenerated rice plants, between 5 and 30 transposed copies of the retrotransposon *Tos17* could be detected, and the number of copies was found to increase with increased time spent in culture (Hirochika et al. 1996).

We have illustrated here that plant genomes are constantly changing in small ways, through errors in DNA replication or spontaneous lesions. They have also changed dramatically on a limited number of occasions in their evolutionary history through polyploidization. These genetic changes are necessary for their survival and adaptation and plants have a remarkable capacity to undergo major genetic change. In addition to these spontaneous genetic changes, plant genomes are further shaped by conventional breeding, which includes the introgression of foreign genes through breeding with wild relatives (Hajjar and Hodgkin 2007; Jones et al. 1995). Genomic comparisons have elegantly illustrated the cumulative impact of conventional breeding on plant genomes. This aspect has been exploited by plant breeders, creating novel varieties in conventional breeding, and recognized by seed production systems that include tolerance for off-types (http://seedgrowers.ca/).

For instance, a comparison of the genomes of 20 rice varieties and landraces determined that the modern indica and japonica varieties each had on average 8.4 Mb of DNA introgressed from other varietal groups and the more traditional aus varieties had an average of 17.5 Mb of introgressed DNA (McNally et al. 2009). In tomato, a study of sequence polymorphisms between fresh-market, processing, vintage and landrace varieties identified lower levels of variation in vintage and landrace varieties that was reflective of the reduced genetic diversity that developed during domestication as a result of genetic bottlenecks (Sim et al. 2009). Broader levels of variation were observed for the fresh-market and processing varieties, reflective of more recent tomato breeding practices in which new genetic variation is introgressed from wild species.

In conclusion, compared to genetic engineering, conventional breeding techniques can have a much larger impact on the plant genome. Hybridization, mutagenesis, and somaclonal variation all give rise to genome-wide genetic change. Similarly, introgression of a desired trait is frequently accompanied by additional unintended traits due to linkage drag, as well as introducing as many, if not more, genetic elements that could influence genes near to the site of introgression, compared to the insertion of DNA by genetic engineering. This premise is supported by several global gene expression profiling studies that illustrate that conventional breeding is more likely to impact the function of the genome than genetic engineering. For instance, mutagenesis of rice induced more changes in gene expression than the insertion of DNA by genetic engineering (Batista et al. 2008). Differences in gene expression are also typically greater between different conventionally bred cultivars than they are between a genetically engineered plant and an untransformed comparator, as demonstrated in wheat and soybean (Baudo et al. 2006; Cheng et al. 2008).

## Genetic changes and risk

As has already been discussed, the relationship between genotype and phenotype is complex and it is also tempered by the environment. Genetic changes may be introduced into plants spontaneously or through conventional breeding or genetic engineering. The buffering capabilities of plant genomes and the quality control systems in plant cells will prevent many of these genetic changes from giving rise to discernible changes in a plant’s phenotype. Even in those cases where genetic changes introduced during conventional breeding or genetic engineering do give rise to unintended traits, the unintended traits may not impact the safety of foods or feeds derived from the plant or the risk to the environment from cultivation of the plant. Any risks will depend on whether the unintended trait has an adverse impact on food or feed safety or the environment, such as production of a novel toxin or a change in weediness, and on how the event containing the unintended trait is developed and processed.

While it is theoretically possible that a new toxin, anti-nutrient or allergen might be introduced into a plant, the potential for this to occur is low. In fact, there are no documented cases where conventional breeding has resulted in the production of a novel toxin or allergen in a crop (Steiner et al. 2013). It is recognized that genetic changes from any type of crop development could alter levels of endogenous toxins or anti-nutrients in plants. However, in most commercially grown crops, these are well characterized and are often monitored to avoid development of crops with negative attributes.

Similarly, while traits that contribute to weediness can be introduced into crop varieties during the conventional breeding process, feral crop populations are rare (Warwick and Stewart 2005), suggesting that the potential for conventional breeding to make a crop weedy is low. This is particularly true for highly domesticated crops that are largely dependent upon cultivation for reproduction.

Another important consideration is how the use of plants—meaning their development, production, storage, processing and preparation—may play a role in managing the risks associated with genetic changes from either genetic engineering or conventional breeding. Cultivar development typically requires upwards of 10 years and involves the evaluation of thousands of plants, resulting in the selection of one or very few final cultivars. Throughout breeding and seed production, selection is applied to eliminate off-types, which are those plants that show an unintended trait. As mentioned above, specific tests may also be conducted on those plants that are known to produce toxins or anti-nutrients in order to eliminate lines that produce unacceptably high levels of such compounds. For instance, glycoalkaloid levels in potato tubers and glucosinolates in canola may be measured. Another important end goal of the selection process is uniformity, which helps to ensure that a cultivar performs consistently once commercially released. The end result of this selection process is that unintended traits which may have occurred are frequently eliminated.

The processing and preparation of foods and feeds may also play a role in managing the risks associated with genetic changes. For example, processing conditions that involve heat or pressure may significantly reduce the levels of toxins and/or anti-nutrients in the food or feed before consumption, so that any genetic changes that alter the levels of such compounds do not present a safety concern. As long as the plants continue to be used in the same way as their conventional counterparts, the risks will be managed to the same extent.

Within the realm of conventional breeding, unintended traits are evaluated primarily for their potential to improve a cultivar. The focus of development for most crops not developed by genetic engineering is on the introduction of desirable characteristics for producers, processors and end-users, without compromising pre-existing characteristics, and while maintaining uniformity. The safety of the genetic changes which occur as a result of conventional breeding is rarely considered, and restricted to known toxins and anti-nutrients. Despite this, examples of adverse effects on human or animal health or the environment are extremely rare, and when they have occurred, they have all involved known compounds, not novel ones (Steiner et al. 2013). There is a history of safe use in agriculture as well as food and feed of cultivars developed using conventional breeding. As a result of this history of safe use, in many countries pre-market assessments are not conducted on new cultivars developed using conventional breeding.

## Conclusions

A number of important conclusions may be drawn from this review of insertional effects and their potential to result in unintended traits. First, insertional effects are an unavoidable consequence of genetic engineering, but the introduction of unintended traits is not. Secondly, a comparison of insertional effects with other genetic changes that occur in plant genomes reveals that genetic changes similar to insertional effects occur spontaneously and during conventional breeding, for example by the movement of transposable elements, the transfer of organelle DNA to the nucleus and as a consequence of the repair of double-strand breaks by NHEJ. A broader consideration of genetic changes that either occur spontaneously or are generated during conventional breeding illustrates that plant genomes are constantly changing (McClintock 1984; Weber et al. 2012), and therefore that insertional effects introduced through genetic engineering make a relatively small contribution to the final genetic make-up of given plant varieties. Finally, the impact of genetic changes in a plant, including but not limited to insertional effects, is influenced by the low potential that these genetic changes will result in a phenotypic change in the plant, and by the low potential for this phenotypic change to have an adverse effect on the plant, or the resulting food and feed safety.

It was hypothesized that if insertional effects are found to be similar to other genetic changes that occur in plants, they should present a similar level of risk. We have illustrated here that the insertional effects associated with genetic engineering are similar to the genetic changes that occur in conventionally bred plants. Based on this similarity, insertional effects should present a similar level of risk as genetic changes associated with conventional breeding. In light of this conclusion, it is reasonable to re-evaluate the information required for the pre-market assessment of genetically engineered plants, foods and feeds. In doing so, it will be important to maintain consistency with international guidelines, such as Codex Alimentarius (2003).

Although these conclusions are relevant to the consideration of all transformation events, a re-evaluation of this information will be particularly useful for retransformants, which are plants that have been transformed with the identical construct(s) as a previously authorized plant of the same species that confers the same trait, and for which the safety of the trait has been previously assessed (CFIA 2012a, b). Retransformants are often generated for vegetatively propagated plants where it can be difficult to transfer a novel trait through sexual crosses, and therefore different cultivars are often independently transformed. Performing individual pre-market assessments on each transformant can be resource-intensive for both crop developers and regulatory authorities. If the genetic elements in the DNA that have been inserted in a retransformant are identical in sequence and order to those inserted in the original transformant, and genes are expressed at a similar level, the only potential difference between the original transformant that was assessed and a retransformant would be insertional effects. In such situations, the conclusions of this paper could be used to inform the pre-market assessment and determine appropriate information requirements.
